# Monitoring data of marine turtles on the Togolese coast during 2012–2013

**DOI:** 10.3897/zookeys.779.26967

**Published:** 2018-08-07

**Authors:** Délagnon Assou, Gabriel H. Segniagbeto, Raoufou Radji, Jacques Akiti, Francisco Pando

**Affiliations:** 1 Department of Zoology, Faculty of Science, University of Lomé, 01BP: 1515, Lomé, Togo University of Lomé Lomé Togo; 2 Togolese Society for the Nature Conservation / Association Togolaise pour la Conservation de la Nature, "ONG AGBO-ZEGUE", BP: 6057, Avepozo – Ibokome, Lomé, Togo Togolese Society for the Nature Conservation Lomé Togo; 3 Laboratory of Forestery Research, Faculty of Science – University of Lomé Laboratory of Forestery Research, Faculty of Science Lomé Togo; 4 Real Jardín Botánico – CSIC, Claudio Moyano 1, 28014, Madrid, Spain Real Jardín Botánico Madrid Spain

**Keywords:** *
Chelonia mydas
*, Cheloniidae, coast, Dermochelyidae, *
Dermochelys coriacea
*, ecoguards, *
Lepidochelys olivacea
*, Togo, West Africa

## Abstract

This dataset contains information on the presence and distribution of sea turtles in Togo. Observations were carried out through a network of ten ecoguards (local guides), facilitated by five fishermen, and coordinated by a field technician, all under the supervision of a scientific coordinator. Data on the occurrence or direct observation of sea turtles on the Togolese coast from September 2012 to August 2013 is presented based on 740 occurrences.

## Introduction

Sea turtles emerged approximately 130 million years ago and are the only marine forms of the reptilian class (Márquez 1990, Frazier 2003). They are migratory species whose populations are essentially distributed in the intertropical zone (Pritchard 1997). Because of their phylogeny, physiology, and behaviour, these species represent an ancient and important component in marine and coastal ecosystems (Ferraroli et al. 2003). They occupy all ecological niches available in the marine ecosystems. They are herbivorous, carnivorous, or omnivorous and are preyed upon by large marine predators such as sharks and orcas (Bjorndal 1997). Frazier (1999) and Bjorndal and Jackson (2003) have demonstrated that these animals play an important role in their habitats, and their vitality depends on the exploitable resources (fish, molluscs, and mangroves). According to Segniagbeto et al. (2017), fishing nets represent the main conservation problem for the various Togolese sea turtle species, and cause demographic strain of turtle populations.

As sea turtles migrate over thousands of kilometres, and the fact that they take tens of years to reach maturity, sea turtles serve as health indicators of coastal and marine environments, both locally and globally (Meylan and Donnelly 1999, Frazier 1999, Fretey 2001). In West Africa and particularly in Togo, a number of studies have focused on marine turtles (Fretey 2001, Segniagbeto et al. 2013, 2014, 2016, 2017). Of the six species known worldwide, five are present in Togo. These are the Green turtle (*Cheloniamydas* (Linnaeus, 1758)), Olive ridley (*Lepidochelysolivacea* (Eschscholtz, 1829)), Loggerhead turtle (*Carettacaretta* (Linnaeus, 1758)), Hawksbill turtle (*Eretmochelysimbricata* (Linnaeus, 1766)), and Leatherback turtle (*Dermochelyscoriacea* (Vandelli, 1761)). According to the above-mentioned works, feeding, and reproduction are the main reasons explaining the presence of these species on the Togolese coast.

As part of the implementation of the Environmental and Social Management Plan (ESMP), linking to the container terminal construction at the Lome Autonomous Port (by Lome Containers Terminal - LCT), a follow-up program for marine turtles was developed between September 2012 and August 2013, to determine the dynamics of their attendance on the Togolese coast. For this purpose, a monitoring protocol has been developed to collect data on the presence of marine turtles at the construction site of the terminal and its area of influence. The objective of this monitoring program was to verify the assumptions made in the ESIA report, which asserts the presence of marine turtles in the project construction zone, and to propose measures to reduce risks of disturbance and accidents of these animals caused by the construction works. The data collected also made possible to analyse the ecological parameters connected to the use of Togolese beaches by marine turtle species. In the following paragraphs, we present the data collection method used in this monitoring program.

**Figure 1.**
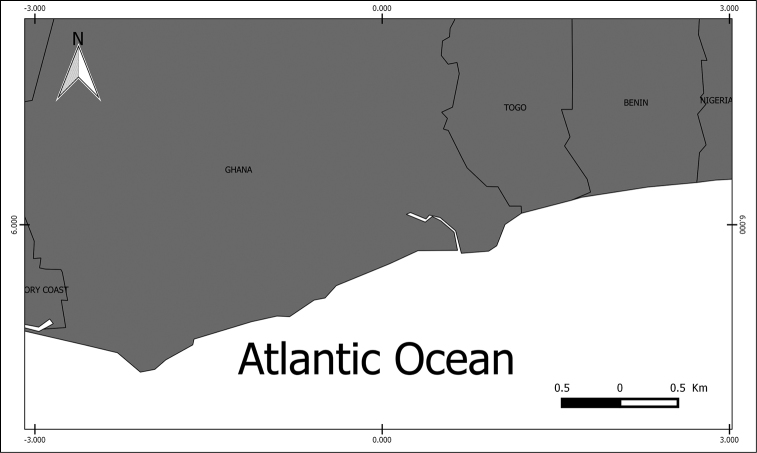
Region of the record Datasets of sea turtles (coastal areas of Ghana, Togo and Benin).

Previous knowledge available in digital form from the GBIF data network is summarized in Table 1 and compared with the data contributed by the dataset described herein, which almost doubles the number of records known for the three species of sea turtles from the region (Figure 1).

**Table 1.** Sea turtle records from the region per species and datasets as available from www.gbif.org (March 2018).

Datasets contributing sea turtle records for the region (Coastal areas of Ghana, Togo and Benin)	Number of records per species			Source
	* L. olivacea *	* C. mydas *	* D. coriacea *	
Tortue Olivâtre. Données publiées dans le cadre du projet JRS Bénin	161	12	25	Dossou-Bodjrènou 2016
Census of the threatened species of Benin.	1	1	1	Kiki and Ganglo 2017
SMNS Herpetologie	1	–	–	Schlüter 2015
iNaturalist Research-grade Observations	1	1	–	iNaturalist.org 2018
National census of *Lepidochelysolivacea* (Benin)	163	12	25	Dossou-Bodjrenon and Dossa Gbo 2015
Census of the animals of Benin	–	1	1	Kingbo and Kiki 2016
Sizing Ocean Giants	–	–	1	McClain and Mackay 2017
Dataset described herein	409	309	19	

## Project details

Project title: BID-AF2015-0004-NAC Monitoring data of marine turtles on the Togolese coast over 2012-2013 years.

Personnel: Délagnon Assou, Gabriel H. Segniagbeto

Data published through GBIF: http://ipt-togo.gbif.fr/resource?r=marine_turtles

### Funding

This marine turtle monitoring program was funded by the LCT (Lome Container Terminal). This company, established in Togo, carried out a project including the design, financing, construction, management, and operation of a private containers terminal at the port of Lomé. In compliance with the setting of the framework environmental law in Togo, an Environmental and Social Impact Assessment (ESIA) with an Environmental and Social Management Plan (ESMP) was carried out before the implementation of the project. Recommendations of this study required a sea turtle monitoring program entrusted to the NGO named AGBO-ZEGUE. Indeed, this NGO is specialized in monitoring populations of endangered marine and coastal species in Togo and West Africa.

Thus, to carry out this monitoring of marine turtles along the Togolese coast, AGBO-ZEGUE has initiated and installed a network of ecoguards whose mission was to collect data on sea turtle attendance and egg-laying.

### Design description

**Figure 2.**
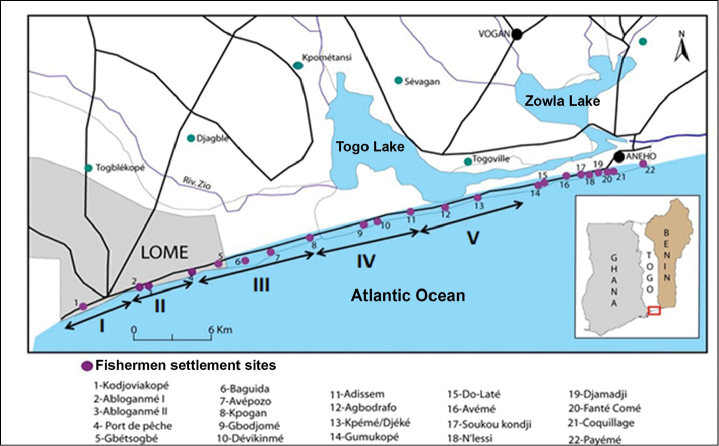
Monitoring program sites (Segniagbeto et al. 2017).

As part of this monitoring program, five observation sites have been defined to cover the Togolese littoral zone where the construction work of the container terminal is likely to impact, in particular on the access of sea turtles to the coast to lay eggs (Figure 2). The description of these sites includes their geographical limits, their physical, topographical and biological characteristics, justifying the motivations that led to their choice.

Site 1: Length = 6 km; Going from the Ghana-Togo border to the Rond-Point of “Hotel de la Paix”. This site presents a sandstone favourable to the nesting of marine turtles. The site is under heavy light disturbance: the lights emitted at night by ships added to the lights of streetlights and buildings can influence the behaviour of egg-laying females.

Site 2: Length = 5.5 km. It extends from “Hotel de la Paix” to the main pier of the autonomous port of Lome. It corresponds to the construction site of the LCT container terminal and is one of the most affected areas by the port activities. The monitoring program took into account this site in order to evaluate the impact of construction activities on its attendance by sea turtles.

Site 3: Length 10 km, from Gbetsogbe village to Kpogan. Like site 2, site 3 is also part of the zone of influence of the construction of LCT. It is the longest site of the monitoring program. It is characterized by a beach-rock bench visible at low tide. This natural building is sometimes a serious handicap for female sea turtles coming to lay at low tide.

Site 4: Length = 8 km, from Kpogan to Adissen village. It is part of the area of influence of the LCT as the site 3. It has the distinction of having a dark beach back at night and therefore conducive to nesting behaviour of sea turtles. The beach-rock bench is present but of low amplitude. This supralittoral consists of sand grain favourable to the incubation of sea turtle eggs. This site is the second most frequented by marine turtles in Togo (Segniagbeto 2004).

Site 5: Length = 8 km, from Adissen to the ore port of Kpémé. It is one of the most frequented sites for sea turtle nesting in Togo (Segniagbeto 2004 and Segniagbeto et al. 2013). Referring to the data on migratory behaviour and local movements of these animals, site 5 is part of the influence zone of construction activities of the container terminal. Indeed, these local displacements are favoured by marine currents that appear on the Togolese coast from West to East. Site 5 offers a dark backside beach at night. Ship lights and human settlements reported on Site 1, would divert these animals to these dark areas. The beach-rock bench is virtually non-existent and thus favours the access of sea turtles to the beach even at low tide. The granulometry of the sand is also favourable to the incubation of sea turtles. Hatchery trials had already been successfully conducted on this site between 2002 and 2005 (Segniagbeto et al. 2013).

## Taxonomic coverage

### General taxonomic coverage description

**Figure 3.**
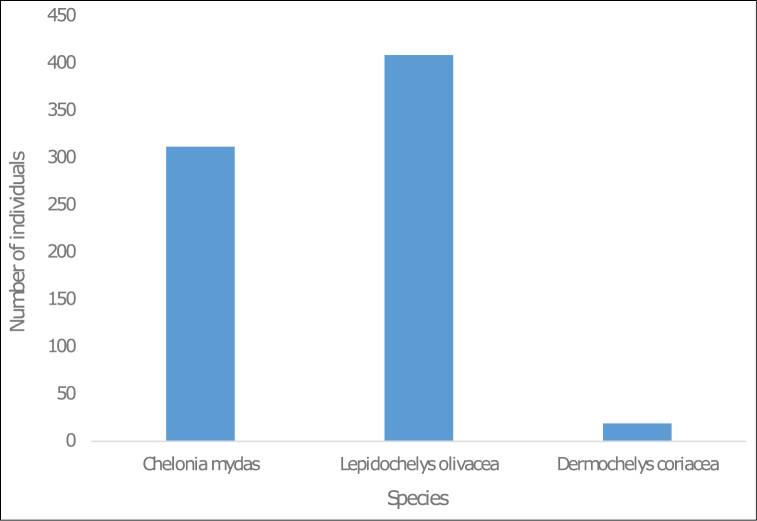
Distribution of marine turtle occurrences by species on the Togolese coast.

**Figure 4.**
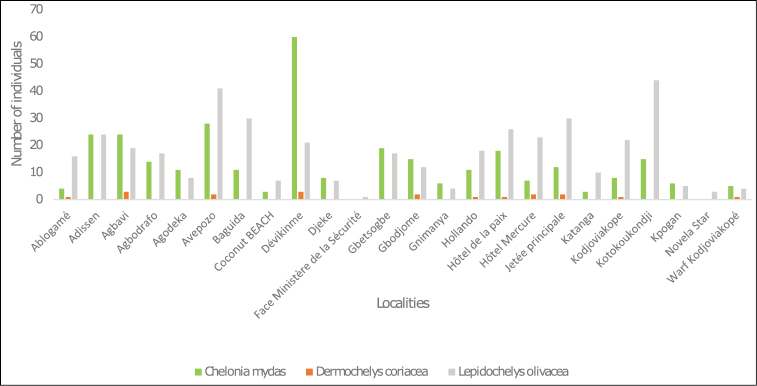
Distribution of marine turtle occurrences by species and location on the Togolese coast.

**Figure 5.**
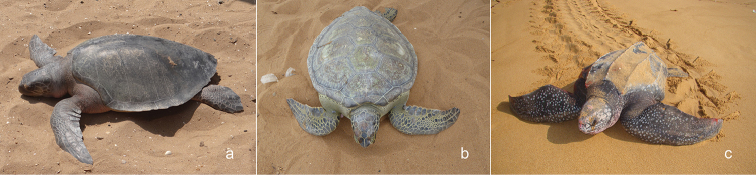
Sea turtle species considered in the survey: **a***Lepidochelysolivacea***b***Cheloniamydas***c***Dermochelyscoriacea*.

During the 2012–2013 period and the monitoring campaign, three species of marine turtles were observed. 740 occurrences were recorded and distributed as follows: 409 individuals of Olive ridley turtle (*Lepidochelysolivacea* (Eschscholtz, 1829)) (Figure 5a), 309 occurrences of Green turtle (*Cheloniamydas* (Linnaeus, 1758)) (Figure 5b), and 19 of Leatherback turtle (*Dermochelyscoriacea* (Vandelli, 1761)) (Figure 5c). Figure 3 shows the distribution of these turtle on the Togolese coast. This distribution is more or less extensive depending on the collection locations (Figure 4).

### Common names

*Lepidochelysolivacea* (Eschscholtz, 1829) (Figure 5a): Olive ridley turtle (English); Eklo (Ewe).

*Cheloniamydas* (Linnaeus, 1758) (Figure 5b): Green turtle (English); Eklo (Ewe).

*Dermochelyscoriacea* (Vandelli, 1761) (Figure 5c): Leatherback turtle (English), Agbo-zegue (Ewe).

### Spatial coverage

General spatial coverage: This monitoring program was carried out all over the Togolese coast. This coast stretches from Kodjoviakopé, the last Togo district before Togo-Ghana border to Aného, a city in Togo which borders Benin (Togo-Benin border). For the study, this area was subdivided into five sites.

Coordinates: The study area is located between 6°6'32.4"N and 6°17'31.2"N Latitude; and 1°8'13.2"E and 1°49'1.2"E Longitude (DMS).

## Methods

Temporal coverage: The monitoring program was implemented from September 2012 to August 2013. During this period, the local guides do the patrols every day from 04:00 to 06:00 am.

### Method step description

**Study extent description**

Monitoring was carried out by a network of ten ecoguards and five facilitator fishers. All activities of ecoguards and facilitators are coordinated by a technical facilitator coming from the NGO. The latter retrieves weekly all data recorded by ecoguards and strips them. In addition to this coordination, he participates in night patrols, in the surveillance of fishermen’s camps during the day and, when necessary, participates in the facilitation of the release of captured sea turtles. Data collected are then transmitted to the NGO scientific coordinator who analyses the data recorded and outputs summary results every semester.

**Sampling description**

The method of data collection is based on regular monitoring of nesting beaches and the different camps of fishermen. Surveillance is organized day and night. For security reasons, patrols at night were organized in the early morning between 04:00 and 06:00 am. Visits were carried out every day from September 2012 to August 2013. During the day, fishing camps are monitored between 10:00 am and 4:00 pm in order to recover and release the animals captured alive by these fishermen. This work is carried out according to the characteristics of the sites in particular the parameters related to the safety of the places and the work period of the coastal fishermen. The average observation effort per ecogard and per site varies between 60 and 90 hours per month. Two ecoguards positioned per site are chosen from the villages located at the ends of their site, and specifically educated for this study patrolled the sections on a daily basis collecting data. During patrols, ecoguards per site meet every day at a point on their site. This technique allows to cover all the highlights of their site. Thus, all information is collected systematically. Each turtle was identified to species and sex, and its carapace was measured with a tape following the technique by Bolten (1999). Data for females coming to lay on these beaches were recorded on a form where details such as species, date and time of laying, the locality, the biometric measurements, the return to sea or the death of the individual were recorded. The form also allowed recording of tracks on beaches, accidental capture and killing by fishermen, killing and other information. Besides this collection form, field notebooks were used to register other observations on each individual encountered on the beaches. Ecoguards used the identification sheets developed by Fretey and Prichard (2000) to recognize the different species present on the Togolese coast.

## Dataset description

**Object name**: Darwin Core Archive BID-AF2015-0004-NAC Monitoring data of marine turtles on the Togolese coast over 2012–2013 years.

**Character encoding**: UTF-8

**Format name**: Darwin Core Archive format

**Format version**: 1.0

**Distribution http://ipt-togo.gbif.fr/resource?r=marine_turtles**

**Publication date of data**: 2018-04-17

**Language**: English

**Licences of use**: Creative Commons Attribution (CC-BY) 4.0 License

**Metadata language**: English

**Date of metadata creation**: 2018-04-17

**Hierarchy level**: Dataset
